# Glycosylation States of Pre- and Post-synaptic Markers of 5-HT Neurons Differ With Sex and 5-HTTLPR Genotype in Cortical Autopsy Samples

**DOI:** 10.3389/fnins.2018.00545

**Published:** 2018-08-10

**Authors:** Jennifer N. K. Nyarko, Maa O. Quartey, Ryan M. Heistad, Paul R. Pennington, Lisa J. Poon, Kaeli J. Knudsen, Odette Allonby, Amr M. El Zawily, Andrew Freywald, Gail Rauw, Glen B. Baker, Darrell D. Mousseau

**Affiliations:** ^1^Cell Signalling Laboratory, Department of Psychiatry, University of Saskatchewan, Saskatoon, SK, Canada; ^2^Department of Pathology and Laboratory Medicine, University of Saskatchewan, Saskatoon, SK, Canada; ^3^Neurochemical Research Unit, Department of Psychiatry, University of Alberta, Edmonton, AB, Canada

**Keywords:** Alzheimer disease, mood disorder, synapse, antidepressant, *SLC6A4*, glycosylation

## Abstract

The serotonin (5-hydroxytryptamine, 5-HT) transporter (5-HTT) gene-linked polymorphic region (5-HTTLPR) is thought to alter 5-HT signaling and contribute to behavioral and cognitive phenotypes in depression as well as Alzheimer disease (AD). We explored how well the short (*S*) and long (*L*) alleles of the 5-HTTLPR align with serotoninergic indices in 60 autopsied cortical samples from early-onset AD/EOAD and late-onset AD/LOAD donors, and age- and sex-matched controls. Stratifying data by either diagnosis-by-genotype or by sex-by-genotype revealed that the donor's 5-HTTLPR genotype, i.e., *L*/*L, S*/*L*, or *S*/*S*, did not affect 5-HTT mRNA or protein expression. However, the glycosylation of 5-HTT was significantly higher in control female (*vs*. male) samples and tended to decrease in female EOAD/LOAD samples, but remained unaltered in male LOAD samples. Glycosylated forms of the vesicular monoamine transporter (VMAT2) were lower in both male and female AD samples, while a sex-by-genotype stratification revealed a loss of VMAT2 glycosylation specifically in females with an *L*/*L* genotype. VMAT2 and 5-HTT glycosylation were correlated in male samples and inversely correlated in female samples in both stratification models. The *S*/*S* genotype aligned with lower levels of 5-HT turnover in females (but not males) and with an increased glycosylation of the post-synaptic 5-HT2C receptor. Interestingly, the changes in presynaptic glycosylation were evident primarily in female carriers of the *APOE* ε4 risk factor for AD. Our data do not support an association between 5-HTTLPR genotype and 5-HTT expression, but they do reveal a non-canonical association of 5-HTTLPR genotype with sex-dependent glycosylation changes in pre- and post-synaptic markers of serotoninergic neurons. These patterns of change suggest adaptive responses in 5-HT signaling and could certainly be contributing to the female prevalence in risk for either depression or AD.

## Introduction

The degeneration of serotoninergic cell bodies in the dorsal raphé nucleus and noradrenergic cell bodies in the locus coeruleus, and their respective ascending projections (Marcyniuk et al., 1986; Zweig et al., 1988; Rub et al., 2000; Parvizi et al., 2001; Grudzien et al., 2007) are acknowledged as critical events in the earliest stages of Alzheimer disease (AD) and likely predispose to a range of physiological and neuropsychiatric sequelae in presymptomatic AD-dementia that potentially are maintained throughout later stages of the disease process.

Serotonin (5-hydroxytryptamine; 5-HT) plays a significant role in cognition, which likely reflects the anatomical association of serotoninergic innervation with brain areas regulating memory and learning (King et al., 2008). Any early-stage changes in monoaminergic tone, compounded by cholinergic deficits, could contribute to the cognitive decline in AD (Robinson, 1983; Richter-Levin and Segal, 1993), while changes in serotoninergic function on its own could be a major contributor to neuropsychiatric symptoms, including depression (Ritchie and Lovestone, 2002). Cortical laminar pathology has been associated with hyperserotoninergic (but not noradrenergic) signaling during the first week of life in mice (Cases et al., 1996) and aberrant serotoninergic signaling usually accompanies cholinergic deficits (Grailhe et al., 2009). Taken together, these observations strongly suggest that serotoninergic dysfunction precedes cholinergic dysfunction and this, in turn, could have significant implications for the order of causality in the monoaminergic-cholinergic dysregulation in AD.

Monoaminergic dysfunction also has been historically associated with depression, a putative risk factor for AD-related dementia (Geerlings et al., 2008; Caraci et al., 2010; Wuwongse et al., 2010). Using provincial (Saskatchewan, Canada) health care utilization data, we reported on a higher risk of mortality in demented male patients with a co-morbid psychiatric disorder compared to demented male/female patients with no psychiatric history (Meng et al., 2012). Depressive symptoms can increase risk of AD/dementia, but, again, selectively in males (Fuhrer et al., 2003), although the *APOE* ε4 genetic risk factor for AD has been associated with increased incidence of depression before onset of AD, but only in females (Delano-Wood et al., 2008). More recently, we reported on the incidence of AD and related dementia being greater in individuals with a history of antidepressant usage, with a moderately higher odds ratio in males (Moraros et al., 2017). Supporting literature implicates the Selective Serotonin Reuptake Inhibitor (SSRI) class of antidepressants in an increased risk of AD (Kessing et al., 2009).

Treatment with SSRIs results in an acute increase in synaptic 5-HT by targeting–and inhibiting–the 5-HT transporter (5-HTT). The 5-HTT is encoded by the *SLC6A4* gene (Gelernter et al., 1995) and a “length polymorphism,” i.e., a 44 bp deletion, in the *SLC6A4* promoter [i.e., the 5-HTT gene-linked polymorphic region: 5-HTTLPR] has been shown–based on luciferase reporter studies–to decrease promoter activity and reduce *5-HTT* mRNA transcription in human placental choriocarcinoma cells (Heils et al., 1996). The short (*S*) allele leads to decreased 5-HTT protein expression [based on [^125^I]-RTI-55 binding in human lymphoblasts] (Lesch et al., 1996) as well as diminished response to SSRIs (Pollock et al., 2000) and increased SSRI side effects (Mundo et al., 2001). Carriers of the *S* allele are also at higher risk of stress-related depressive symptoms and suicidality (Caspi et al., 2003), severe depression (Cervilla et al., 2006), anxiety-like behaviors (Lesch et al., 1996), and eating disorders (Calati et al., 2011). Moreover, this same allele has been associated with cognitive deficits in depressed individuals (Kalska et al., 2013) and in older adults (Garrett et al., 2015), and has been proposed as a risk factor for AD/dementia (Li et al., 1997; Oliveira et al., 1998), although a later study was unable to corroborate any associated risk in a Japanese AD cohort (Kunugi et al., 2000).

The influence of 5-HTTLPR genotype on depressive phenotype remains contentious. There is a reasonable consensus that the *S* allele is closely associated with diminished 5-HT, and while [^125^I]-RTI-55 binding in lymphoblasts (Lesch et al., 1996) as well as [^123^I]-CIT SPECT experiments (Heinz et al., 2000) support the association of the *S* allele with lower expression of the 5-HTT protein, other groups–one of which also used [^123^I]-CIT SPECT–were unable to confirm this association (Greenberg et al., 1999; van Dyck et al., 2004). In fact, the latter study observed a higher, rather than lower, expression of 5-HTT in *S*/*S* individuals (van Dyck et al., 2004), which was subsequently corroborated using [^11^C]-DASB PET in a cohort of 42 healthy adult males (Bose et al., 2011). A meta-analysis was not able to support a role for 5-HTTLPR length polymorphisms, i.e., *S* and Long (*L*) alleles, in predicting antidepressant response and/or remission rates (Taylor et al., 2010), further adding to the contention. Sex-dependent phenotypes must be considered here as it is known that depressed women tend to respond better to inhibitors of monoamine oxidase (MAO), whereas men tend to respond better to uptake inhibitors (Davidson and Pelton, 1986). Furthermore, female carriers of the *S* allele tend to have smaller hippocampal volume (an observation relevant to both depression as well as AD), whereas male carriers of the allele have significantly lower hippocampal volume only if they have experienced an adverse childhood event (Everaerd et al., 2012). The contention extends to the influence of the 5-HTTLPR genotype on *5-HTT* mRNA expression. For example, while luciferase reporter studies support a higher activity of the 5-HTTLPR *L* allele (Heils et al., 1996) and increased *5-HTT* mRNA transcript levels have been detected in the raphé nuclei of *L*/*L* individuals (Little et al., 1998), a study based on human pons as well as B lymphocytes was unable to detect any change in *5-HTT* mRNA transcript levels, regardless of the donor's 5-HTTLPR genotype (Lim et al., 2006).

The fact that overall monoaminergic systems are affected in the AD brain is clear. What remains unclear is how the 5-HTTLPR *S* and *L* alleles align with serotoninergic indices in the male and female brain, and how this might inform on risk and/or a diagnosis of AD. Our current sample set implicates cortical serotoninergic dysfunction in the AD brain or in carriers of the long allele of the 5-HTTLPR. While 5-HTTLPR allelic variants do not align readily with 5-HTT expression, they do align with changes in the glycosylation status of several synapse-associated proteins. The loss of glycosylation is known to occur in many disease states (Ohtsubo and Marth, 2006) and our observed loss of glycosylation of VMAT2 (a marker of monoaminergic neurons) concurrent with a relative increase in the glycosylated 5-HT2C receptor (i.e., active, membrane-associated) support the complexity of glycosylation events observed in AD (Frenkel-Pinter et al., 2017). Some of these glycosylation changes in our sample set are sex-dependent and could be reflecting adaptive responses designed (Heiming and Schaser, 2010) to overcome signaling deficits during the course of the disease.

## Experimental procedures

### Reagents and antibodies

The anti-5-HTT antibody (H-115: sc-13997) and total GSK-3α/β antibody (sc-7291) were obtained from Santa Cruz Biotechnology (Santa Cruz, CA, USA). The anti-SNAP25 antibody (SMI-81R) was obtained from BioLegend (San Diego, CA, USA). The anti-VMAT2 [ab191121], anti-PSD-95 [ab76115], and anti-5-HT2C receptor [EPR6487: ab133570] antibodies were purchased from Abcam Inc (Toronto, ON, Canada). The anti-mouse/anti-rabbit IgG-HRP conjugates were obtained from Bio-Rad Laboratories (Canada) Ltd. All other reagents were obtained from commercial sources.

### Human brain samples

Autopsied cortical samples matched as close as possible for age and sex were obtained from the Douglas-Bell Canada Brain Bank (McGill University, Montréal, Canada). These included 26 male (M) and female (F) controls (CTL: 12M/14F); 16 early-onset AD (EOAD: 7M/9F); and 18 late-onset/sporadic AD (LOAD: 8M/10F). All AD donors had a neuropathological diagnosis according to the CERAD criteria, confirmed by on-site pathologists and based on staining with Hematoxylin and Eosin, modified Belschowsky, and alkaline Congo red. Our samples represent a mix of middle and superior frontal cortices (Brodmann Areas 46/9, respectively) and were chosen as they represent areas with relative hypoperfusion in AD patients with co-morbid depression (Levy-Cooperman et al., 2008). These experiments are covered by the University of Saskatchewan's Research Ethics Office Certificate of Approval: Bio 06-124 (DDM: Principal Investigator).

### 5-HTTLPR genotyping

Genomic DNA was extracted (Wu et al., 1995) and used for amplification of the 5-HTTLPR polymorphic region, i.e., the promoter region of *SLC6A4* spanning the 44 bp deletion (Ehli et al., 2008), with modification. Briefly, 50 ng of DNA was incubated with 300 μM of each dNTP, 0.4 μM of each primer in a final volume of 50 μL using the 5′-ATG CCA GCA CCT AAC CCC TAA TGT and 3′-GGA CCG CAA GGT GGG CGG GA primer-pair. Thermocycling (35 cycles of 94°C/30 s; 66°C/30 s; 72°C/40 s) resulted in amplicons of 419 and 375 bp that were resolved on 2% agarose and visualized using Gel Red stain/UV.

### Quantitative real-time PCR (qPCR)

Total RNA was isolated using an RNeasy® Mini Kit (Qiagen; Mississauga, ON, Canada) and reverse-transcribed to cDNA using SuperScript™ RNase H-Reverse Transcriptase (Invitrogen). cDNA was quantified by nanodrop spectrophotometry and used for qPCR analysis. Gene expression was quantified using the Taqman® primers, labeled probe system, and an ABI 7300 thermocycler from Applied Biosystems (Foster City, CA, USA). Reactions (10 ng of cDNA) were performed using the Taqman Gene Expression assays for three FAM-labeled primer pairs for *SLC6A4* cDNA spanning splice sites between exons (Ex) 3-4 (Hs00984354_m1), Ex8-9 (Hs00169010_m1), and Ex12-13 (Hs00984349_m1). Thermocycling parameters were as follows: 2 min at 50°C, 10 min at 95°C, 40 cycles of 15 s at 95°C/70 s at 60°C. All comparisons [with 3–4 replicates per sample] were performed using quantification software (Applied Biosystems).

### Immunodetection

Tissue homogenates were precleared by centrifugation (12,000 × *g*, 10 min, 4°C) and used for protein determination based on the Lowry (Folin-Ciocalteu reagent) assay. This assay was performed by a single individual so as to decrease inter-sampling variability of our limited tissue samples. Aliquots of proteins (15 μg/lane) were resolved under standard SDS-PAGE denaturing conditions and transferred to nitrocellulose for immunoblotting (Cao et al., 2009; Wei et al., 2012). Detection relied on enhanced chemiluminescence and ImageJ 1.32j (http://rsb.info.nih.gov/ij/) was used for semiquantitative densitometric analyses of scanned blots. The expression of total GSK-3α/β was used to monitor protein loading across samples.

### High pressure liquid chromatography (HPLC)

Concentrations of serotonin (5-hydroxytryptamine; 5-HT) and dopamine (DA), and their respective acid metabolites, i.e., 5-hydroxyindole-3-acetic acid (5-HIAA) and homovanillic acid (HVA), were determined by HPLC by comparing peak height ratios of analytes to those of a set of authentic standards processed in parallel as previously reported (Wei et al., 2012).

### Immunohistochemistry and confocal microscopy

A free-floating protocol as described previously (Chlan-Fourney et al., 2011) was used. Briefly, the section was incubated in citrate buffer for antigen retrieval, blocked with 10% fetal bovine serum, and probed for 5-HTT (1:30 dilution; H-115 antibody). It was then incubated with biotinylated donkey anti-rabbit (1:200 dilution; Vector Laboratories Canada; Burlington, ON, Canada) and processed by the avidin–biotin–peroxidase/3,3′-diaminobenzidine (DAB) method. 5-HTT immunodetection in fixed-cells was done as described elsewhere (Allonby et al., 2014). Briefly, cells were plated on glass-bottom culture dishes, fixed in formaldehyde/PBS, permeabilized, and blocked in PBS containing normal horse serum (Sigma-Aldrich, Oakville, ON, Canada). Alexafluor-conjugated antibody was used for detection. ProLong™ Gold Antifade Mountant with DAPI (P36931) (Invitrogen; Burlington, ON, Canada) provided nuclear staining. Cells were visualized using 60X oil immersion on an Olympus FV1000 confocal microscope. Images were deconvoluted using Auto-Deblur (AutoQuant X3, Media Cybernetics) and processed with the Image J software.

### *APOE* genotyping

*APOE* restriction isotyping for the two single nucleotide polymorphisms–i.e., rs429358 (APOE-C112R) and rs7412 (APOE-R158C)–was done as described elsewhere (Nyarko et al., 2018b). Briefly, 500 ng of genomic DNA was subjected to PCR and the resulting 226 bp amplicon was restricted with *Afl*III and *Hae*II. The fragments were resolved on a 10% non-denaturing, polyacrylamide gel and visualized by staining with GelRed (Biotium). Genotyping identified allelic homo- and hetero-zygotes, with the frequency of ε4 carriers across cases supporting the literature (Poirier et al., 1993).

## Statistical analyses

Any possibility of bias using our autopsy-derived data was minimized by having some individuals assay de-identified samples (i.e., qPCR, Western blotting) and others perform the analysis, i.e., densitometry on scanned images. Data were analyzed using non-parametric models, i.e., either the Mann-Whitney *U* test or ANOVA (Kruskal-Wallis) with adjustment for multiple comparisons using Dunn's *post hoc* test. Significance was set at *P* < 0.05, but values that fell between 0.051 and 0.099 were discussed as *tendencies*. Data are represented as scatter plots with the line representing the sampling mean. Control (CTL) donor sample means were used as the comparator when analyzing for diagnosis-related patterns; the *S*/*S* genotype was used as the comparator when analyzing for any 5-HTTLPR genotype-related patterns. Due to space limitations and for purposes of clarity, analyses that do not reach statistical significance might be simply referred to as “*data not shown*.” We acknowledge that a limitation in the interpretation of our data is that our 60 sample set was not sufficiently powered to undertake relevant three-way stratification, i.e., sex-by-diagnosis-by-genotype. Correlation statistics were based on Pearson's correlation coefficient (r).

## Results

### Donor statistics

The post-mortem interval did not differ between control, EOAD and LOAD samples (*P* = 0.5697). As expected, the age of disease onset [*P* < 0.0001] and the age of the donor at autopsy (*P* < 0.0001) was significantly different between EOAD and LOAD donors, while the brain weight was impacted by a diagnosis of EOAD/LOAD (*P* < 0.0001) (Table 1).

**Table 1:** Basic donor parameters.

		**Control (26)**	**EOAD (16)**	**LOAD (18)**
Sex	M/F	12/14	7/9	8/10
Age, years	M+F	70.73 ± 12.49	58.13 ± 7.80^*^	83.17 ± 5.77^**^
	M	70.67 ± 9.85	63.14 ± 5.61	82.88 ± 5.38^*^
	F	70.79 ± 14.76	54.22 ± 7.16^*^	83.40 ± 6.35
PMI, h	M+F	19.26 ± 10.98	21.44 ± 10.42	21.41 ± 9.06
	M	16.66 ± 8.50	25.21 ± 10.58	22.66 ± 8.54
	F	21.67 ± 12.73	18.51 ± 9.87	20.40 ± 7.98
Brain weight, g	M+F	1232 ± 136.5	1016 ± 200.1^**^	1036 ± 121.9^***^
	M	1275 ± 155.1	1157 ± 139.2	1126 ± 98.9^*^
	F	1191 ± 108.6	879.4 ± 143.4^***^	992.0 ± 94.9^*^
*5-HTTLPR*		*S*/*S*; *S*/*L*; *L*/*L*	*S*/*S*; *S*/*L*; *L*/*L*	*S*/*S*; *S*/*L*; *L*/*L*
	M+F	5; 16; 5	2; 7; 7	2; 7; 9
	M	1; 9; 2	0; 4; 3	2; 3; 3
	F	4; 7; 3	2; 3; 4	0; 4; 6

*EOAD, early-onset Alzheimer disease; LOAD, late-onset Alzheimer disease; M, male; F, female; years (age at autopsy); PMI, post-mortem interval; h, hour; g, gram*;

* *P < 0.05*;

** *P < 0.01*;

*** *P < 0.001; P-values are reported vs. control donors*.

### (a) Serotoninergic indices stratified by diagnosis and sex

#### 5-HTT protein expression

**Figure 1:**
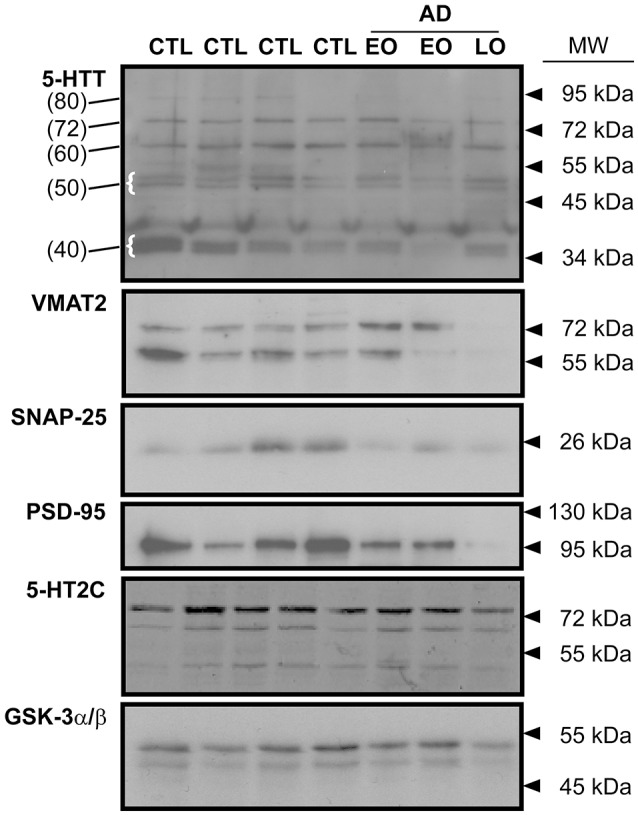
Representative immunoblots of key indicies of the 5-HT synapse in human frontal cortex. The expression of the 5-HT transporter (5-HTT), the vesicular monoamine transporter (VMAT2), the presynaptic marker, SNAP-25, the post-synaptic marker, PSD-95, and the post-synaptic 5-HT2C receptor are shown. Levels of protein loading were monitored by probing for GSK-3α/β. CTL: control; EO (early-onset) and LO (late-onset) AD (Alzheimer disease). In the 5-HTT panel, the numbers in parentheses (at left) identify the molecular weights of specific 5-HTT bands (some highlighted with curly brackets) that were used for densitometry and analysis. Arrowheads represent molecular weights of protein standards/ladders.

The immunodetection of 5-HTT using Western blotting revealed several distinct species, notably a band at ~ 80–90 kDa (presumed glycosylated; referred to herein as 80 kDa), a ~72 kDa band (the mature protein; 72 kDa), a band at ~55–62 kDa (unglycosylated; referred to as 60 kDa) (Tate and Blakely, 1994; Ozaslan et al., 2003). Several smaller bands, i.e., a doublet at ~50 kDa and bands (e.g., ~37–40 kDa: referred to as 40 kDa) (Dmitriev et al., 2005) were also detected (Figure 1).

**Figure 2:**
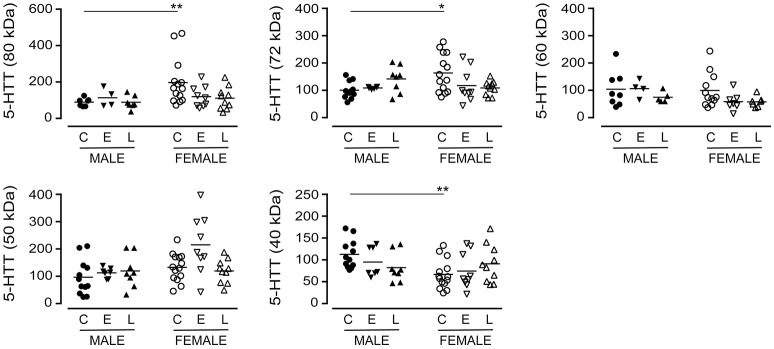
Levels of 5-HTT were examined in control (C), Early-Onset AD (E), and Late-Onset AD (L) samples by densitometric analysis of western blots immunoblotted with anti-5-HTT antibody. Samples were separated according to sex. Data are expressed in relative units. *P < 0.05; **P < 0.01 between indicated groups.

The immunodetection of the 80 kDa (*P* = 0.1843), the 72 kDa (*P* = 0.8507) or the 60 kDa (*P* = 0.2519) bands in pooled (i.e., male+female) samples did not vary with a diagnosis of EOAD or LOAD (*data not shown*). When the data were separated by sex of the donor (Figure 2), a difference in basal expression between male and female controls was revealed for the 80 kDa (*P* = 0.0074) and 72 kDa (*P* = 0.0173) bands, but not for the 60 kDa band (*P* = 0.8994). Furthermore, while not reaching significance, there were tendencies for decreases with the 80 kDa (*P* = 0.0691) and 72 kDa (*P* = 0.0992) bands in females with EOAD and LOAD, and a modest increase in the 72 kDa band in males with LOAD (*P* = 0.0730). Levels of the 50 kDa 5-HTT were not altered across sex/diagnosis, but a significantly lower level of the 40 kDa species was observed in CTL female samples *vs*. CTL male samples (*P* = 0.0013) (Figure 2).

#### VMAT2, SNAP-25, and PSD-95 expression

**Figure 3:**
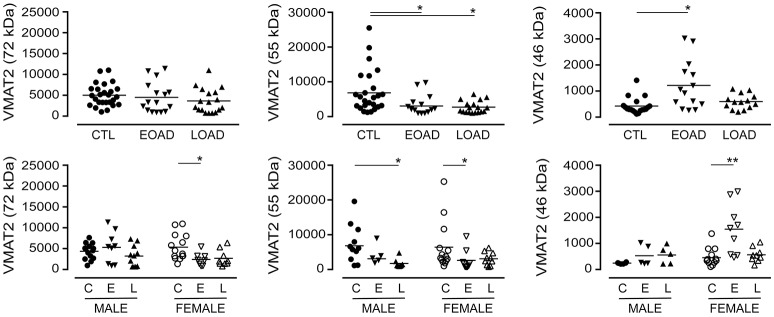
Levels of VMAT2 were determined in pooled (male+female) control (CTL; C), Early-Onset AD (EOAD; E), and Late-Onset AD (LOAD; L) samples by densitometric analysis of probed western blots. (**bottom**) The data were separated according to sex. Data are expressed in relative units. *P < 0.05; **P < 0.01 between indicated groups.

The expression of VMAT2 is a marker of monoaminergic neuronal integrity (Henry et al., 1998) that has been shown to be altered in the AD brain (Lehericy et al., 1994). The VMAT2 protein can be detected as a 72 kDa (glycosylated), a 55 kDa (partially glycosylated), and a 46 kDa (mature) species (Cruz-Muros et al., 2008). In our samples, the glycosylation of VMAT2 was not altered in AD samples (*P* = 0.1987), although stratification for sex revealed a significant decrease in female EOAD samples (*P* = 0.0489) (Figure 3). The partially glycosylated form (*i.e*. 55 kDa) of VMAT2 was decreased in both EOAD and LOAD samples (*P* = 0.0047) with contributions from both sexes. There was an increase in the unglycosylated form of VMAT2 in the EOAD samples (*P* = 0.0048), which aligned primarily with an increase in female EOAD samples (*P* = 0.0027) (Figure 3).

**Figure 4:**
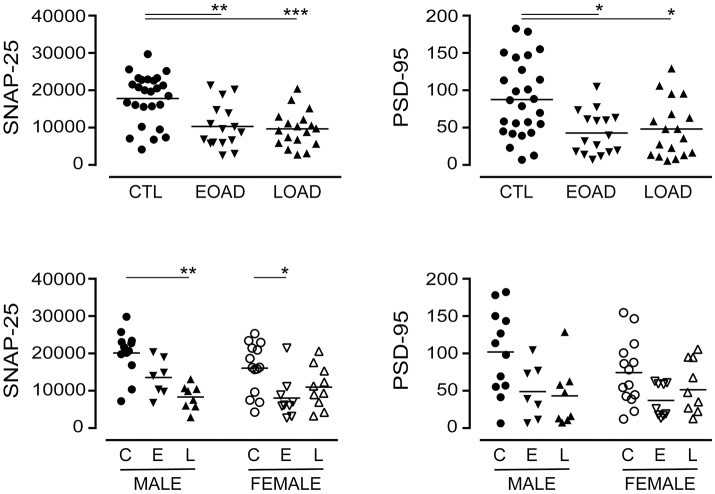
Levels of SNAP-25 and PSD-95 were examined in pooled (male+female) control (CTL; C), Early-Onset AD (EOAD; E), and Late-Onset AD (LOAD; L) samples by densitometric analysis of probed western blots. (**bottom**) The data were separated according to sex. Data are expressed in relative units. *P < 0.05; **P < 0.01; ***P < 0.0001 between indicated groups.

We also included markers of synaptic integrity in our analyses. The levels and/or expression of the presynaptic marker SNAP-25 (Greber et al., 1999; Brinkmalm et al., 2014) and the postsynaptic marker PSD-95 (Savioz et al., 2014) have been found to be altered in the AD brain. In our sample set, there was a significant loss of SNAP-25 expression in cortical EOAD and LOAD samples (*P* = 0.0002), with contributions from both male and female AD donors (Figure 4). There was also a significant loss of PSD-95 expression in pooled EOAD or LOAD samples (*P* = 0.0084), but any significance was lost (due to variability within groups) when stratifying the data by sex (Figure 4).

### (b) 5-HTT protein stratified by diagnosis and 5-HTTLPR allelic variant

#### 5-HTTLPR genotypes in our sample set

**Figure 5:**
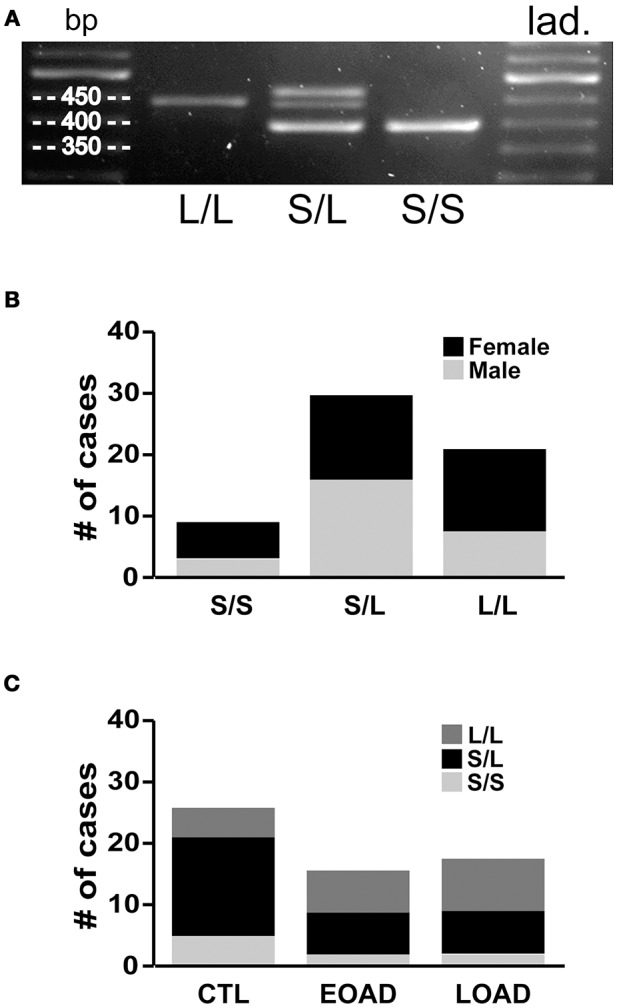
5-HTT genotyping. **(A)** Standard PCR was used to amplify the 5-HTTLPR (spans the region of the promoter in which a 44 bp sequence is present or deleted) and amplicons were resolved on 2% agarose gel to determine whether the sample contains only the long allele *(L*/*L*: 419 bp), only the short allele (*S*/*S*: 375 bp), or both (*S*/*L*). **(B)** The frequency of the allelic variants (i.e., *S*/*S, S/L* and *L*/*L*) in our 60 brain samples. **(C)** The distribution was as expected in the control (CTL) samples; however, the Early-Onset AD (EOAD) or Late-Onset AD (LOAD) samples were not associated with any significantly higher number of the *S* allele.

We re-examined the data based on the donor's 5-HTTLPR genotype, i.e., *S*/*S, S*/*L*, or *L*/*L* status. Our 60 samples included 15% *S*/*S* (9/60), 50% *S*/*L* (30/60), and 35% *L*/*L* (21/60) (Figures 5A,B), which is remarkably similar to the distribution of 5-HTTLPR allelic variants published in the original report by Lesch and colleagues, i.e., 19, 49, and 32%, respectively (Lesch et al., 1996). The three genotypes were represented between the sexes, although this distribution–limited to 60 donor tissues–did not allow for a relevant three-way stratification analysis, i.e., sex-by-diagnosis-by-genotype, as some of our groups do not have sufficient sample size. For example, EOAD donors, who are also carriers of two *S* alleles (Figure 5C), would only be represented by a sample size of *n* = 2 females. Based on this limitation, we chose to examine the two possible two-way stratifications based on genotype, i.e., (a) diagnosis-by-genotype; and (b) sex-by-genotype.

### Serotoninergic indices stratified by diagnosis and 5-HTTLPR genotype

#### *5-HTT* mRNA

Luciferase reporter gene assays, based on studies in human JAR cells (Heils et al., 1996) and lymphoblasts (Lesch et al., 1996), suggest that the 44 bp deletion in the 5-HTTLPR *S* allele significantly hinders cAMP- and protein kinase C-dependent transcriptional activity. We examined our samples for *5-HTT* mRNA transcript levels based on primer-pairs spanning the acceptor-donor splice sites for Ex3–4, Ex8–9, and Ex12–13. *5-HTT* mRNA transcript levels [Ex3–4 (*P* = 0.7020); Ex8–9 (*P* = 0.1631); Ex12–13 (*P* = 0.0706)] were not significantly different between controls and a diagnosis of EOAD or LOAD (*data not shown*). *5-HTT* mRNA transcript levels [Ex3–4 (*P* = 0.7513); Ex8–9 (*P* = 0.2390); Ex12–13 (*P* = 0.5263)] were not significantly different between *S*/*S, S*/*L*, and *L*/*L* genotypes (*data not shown*).

#### 5-HTT, VMAT2, SNAP-25, and PSD-95 expression based on diagnosis-by-genotype stratification

This stratification model did not reveal any significant change in 5-HTT protein species in any of the groups (the *P*-values ranged from 0.351 to 0.963, *data not shown*). In addition, this stratification did not reveal any significant changes or trends in VMAT2, SNAP-25, or PSD-95 levels (*data not shown*).

*A priori*, the lack of an interaction between 5-HTTLPR genotype (i.e., *S* and *L* alleles) and a diagnosis of AD on *5-HTT* mRNA transcription, on 5-HTT glycosylation/expression, and on the levels of synaptic markers of AD progression (i.e., SNAP-25, PSD-95) in our sample set do not support the reported differences in risk of developing AD ascribed to these 5-HTTLPR length polymorphisms.

### (c) Serotoninergic indices stratified by sex and 5-HTTLPR genotype

#### 5-HTT expression

**Figure 6:**
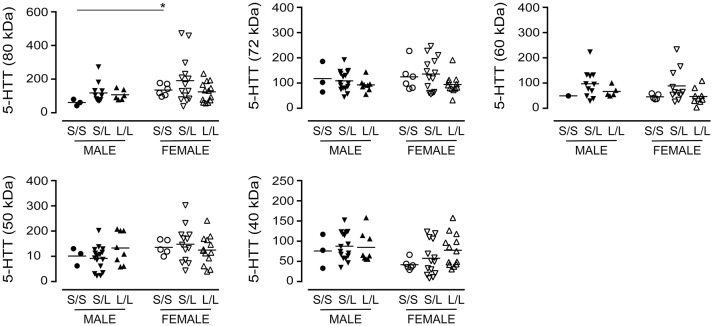
The levels of 5-HTT [using the data (relative units) presented in Figure 2 ] were stratified based on the donor's sex and 5-HTTLPR genotype (*S*/*S, S*/*L*, or *L*/*L*), and ignoring diagnosis. *P < 0.05 between indicated groups.

We re-examined our data based on sex-by-genotype (i.e., excluding “diagnosis of AD” as a nominal variable). Levels of the various 5-HTT species (*P* values ranging from 0.1273 to 0.7087) were not altered in 5-HTTLPR genotype, i.e., *S*/*S, S*/*L*, or *L*/*L*. Aside from a significantly higher level of 5-HTT glycosylation (e.g., 80 kDa species) in females with an *S*/*S* genotype *vs*. males with an *S*/*S* genotype (*P* = 0.0238) (Figure 6), there was no generalizable effect of 5-HTTLPR genotype on any of the 5-HTT species when stratified by sex.

#### VMAT2, SNAP-25, and PSD-95 expression

**Figure 7:**
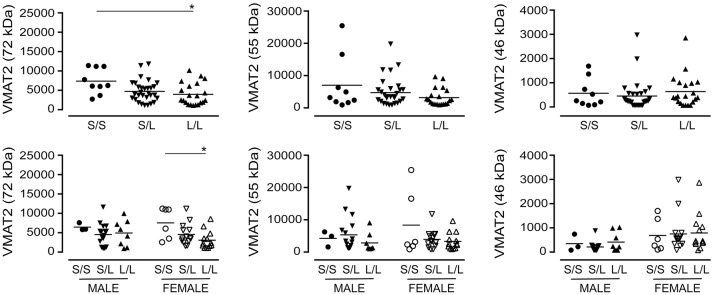
The levels of VMAT2 [using the data (relative units) presented in Figure 3] were stratified based on the donor's sex and 5-HTTLPR genotype (*S*/*S, S*/*L*, or *L*/*L*), and ignoring diagnosis. *P < 0.05 between indicated groups.

**Figure 8:**
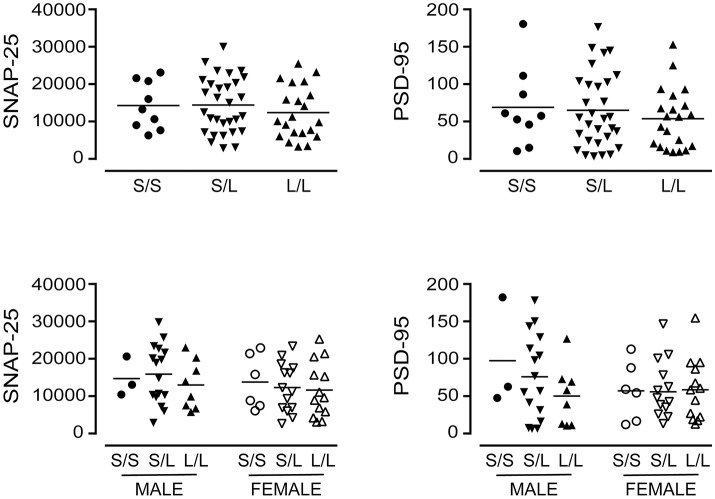
The levels of SNAP-25 and PSD-95 [using the data (relative units) presented in Figure 4] were re-stratified based on the donor's sex and 5-HTTLPR genotype (*S*/*S, S*/*L*, or *L*/*L*), and ignoring diagnosis. There were no significant changes observed.

A significant decrease in glycosylated VMAT2 (*P* = 0.0252) was observed in *L*/*L* samples, which aligned with changes specifically in female *L*/*L* samples (*P* = 0.0232) (Figure 7). In contrast, levels of the partly glycosylated VMAT2 species (*P* = 0.3296) and the unglycosylated VMAT2 (*P* = 0.3319) were not altered by 5-HTTLPR genotype, regardless of sex/genotype. Levels of SNAP-25 (*P* = 0.5121) and PSD-95 (*P* = 0.7582) were also not affected by 5-HTTLPR genotype (Figure 8).

#### 5-HTT and VMAT2 glycosylation are inversely correlated

**Figure 9:**
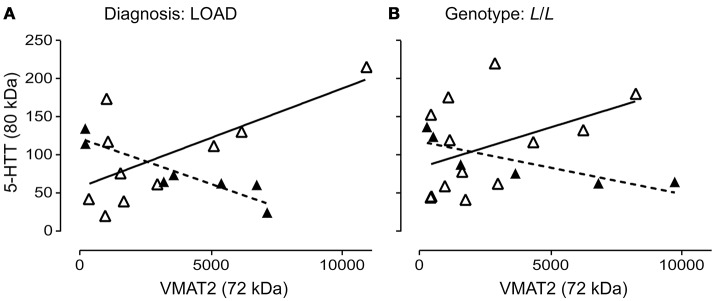
Pre-synaptic proteins in the 5-HT synapse are differentially glycosylated in males and females. **(A)** The correlation of glycosylated VMAT2 (72 kDa species) and glycosylated 5-HTT (80 kDa species) in males (*r* = 0.9248) with late-onset AD (▴), dashed line and in females (*r* = 0.6863) with late-onset AD (Δ), solid line. **(B)** The correlation of glycosylated VMAT2 and glycosylated 5-HTT in males (*r* = 0.8396) with a 5-HTT *L*/*L* genotype (▴), dashed line and in females (no correlation) with a 5-HTT *L*/*L* genotype (Δ), solid line.

The 5-HTT and VMAT2 are both expressed on the 5-HT terminal. We wondered whether their glycosylation states might be correlated. The glycosylation states of 5-HTT (80 kDa) and VMAT2 (72 kDa) were negatively correlated in males (*P* = 0.0029; *r* = 0.9248) and positively correlated in females (*P* = 0.0284; *r* = 0.6863) with a diagnosis of LOAD (Figure 9A). In contrast, while the glycosylation states of 5-HTT and VMAT2 were also negatively correlated in males with an *L*/*L* genotype (*P* = 0.0365; *r* = 0.8396), there was no correlation between the levels of the two glycosylated proteins in females with the *L*/*L* genotype (*P* = 0.1431) (Figure 9B). Correlations were not significant in controls or EOAD samples, or in 5-HTTLPR *S*/*S* and *S*/*L* carriers (when stratified by genotype).

#### Levels of 5-HT and dopamine (DA) stratified by diagnosis and/or 5-HTTLPR genotype

**Figure 10:**
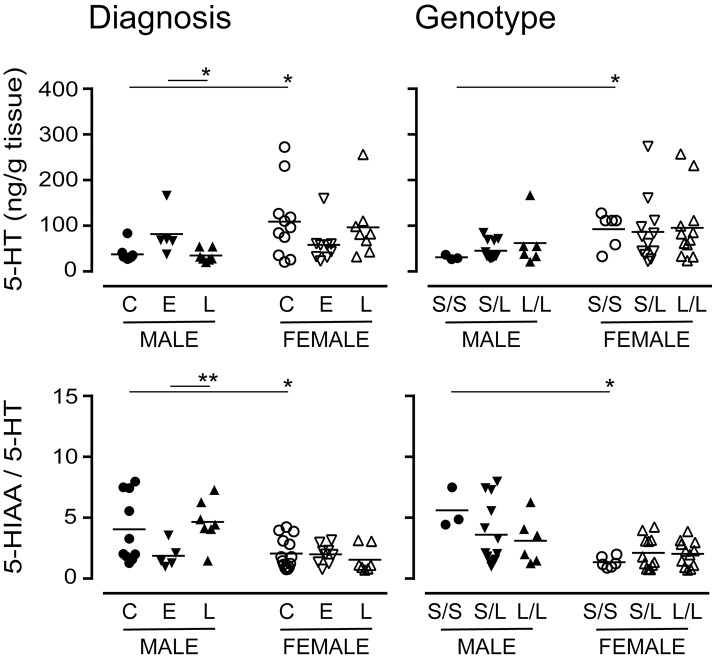
Levels of 5-HT and its major acid metabolite, 5-HIAA, were measured by HPLC analysis. The 5-HIAA/5-HT ratio is used as an index of 5-HT turnover. Data were separated according to the donor's sex and diagnosis [control (C), Early-Onset AD (E), and Late-Onset AD (L)] or sex and 5-HTTLPR genotype (*S*/*S, S*/*L* or *L*/*L*). *P < 0.05, **P < 0.01 between indicated groups.

The 5-HTTLPR genotype is thought to alter 5-HT availability (Lesch et al., 1996). There were significantly higher levels of 5-HT (the substrate for 5-HTT) in female control or *S*/*S* samples vs. the corresponding male samples (Figure 10). While there were significant differences in 5-HT levels in males with a diagnosis of EOAD *vs*. LOAD, 5-HT levels in females were not significantly altered, regardless of diagnosis or genotype. The 5-HIAA/5-HT ratio, an indicator of 5-HT turnover, was significantly lower in female samples (control or *S*/*S*) *vs*. the corresponding male samples (Figure 10). The change in turnover in this stratification was independent of any change in the activity of the MAO-A enzyme that converts 5-HT to 5-HIAA (*data not shown*). In contrast, levels of DA (*P* = 0.8113) or its metabolite HVA (*P* = 0.5454), and the HVA-to-DA ratio (*P* = 0.4307) were not affected by diagnosis or by 5-HTTLPR genotype (DA: *P* = 0.1034; HVA: *P* = 0.2469; HVA-to-DA: *P* = 0.3078). These trends were not altered by sex in either stratification (*data not shown*).

**Figure 11:**
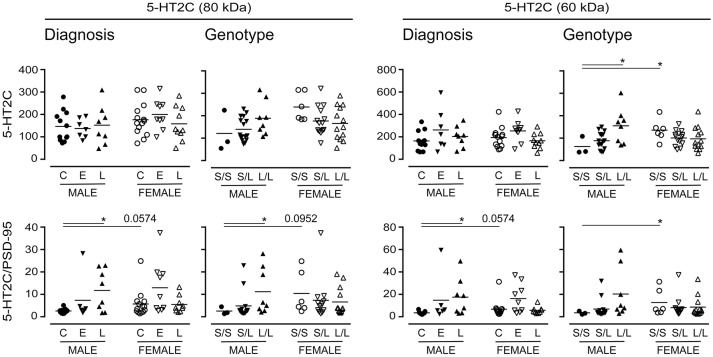
Levels of the post-synaptic 5-HT2C receptor were stratified by diagnosis or by 5-HTTLPR genotype. Data (relative units) represent control (C), Early-Onset AD (E), and Late-Onset AD (L) donors or the allelic variants (i.e., *S*/*S, S/L*, and *L*/*L*) and were separated according to sex. (**bottom**) The same data were normalized to the expression levels of the post-synaptic marker, PSD-95. *P < 0.05 between indicated groups.

Long-term changes in the synaptic availability of 5-HT can regulate changes in the post-synaptic signaling receptor population. As such, we chose to examine the expression of the post-synaptic 5-HT2C receptor (Anastasio et al., 2010), which also has a number of glycosylation states (Backstrom et al., 1995). There were no significant changes in 5-HT2C receptor expression when data were stratified for “diagnosis,” yet emerging tendencies were apparent when the samples were stratified for 5-HTTLPR genotype; for example, immunodetection of the 60 kDa 5-HT2C receptor increased in males with an *L*/*L* genotype (*vs. S*/*S*) (*P* = 0.04) (Figure 11). Given its post-synaptic localization and the fact that 5-HT2C receptor is known to interact with the PSD-95 marker of synaptic integrity (Anastasio et al., 2010), we normalized the 5-HT2C receptor expression data to PSD-95 expression; this revealed significant increases in relative 80 kDa 5-HT2C receptor expression in males with a diagnosis of LOAD (vs. male controls) and in males with an *L*/*L* genotype (*vs*. males with an *S*/*S* genotype) (Figure 11). An increase was also observed in the detection of the 60 kDa 5-HT2C receptor species in LOAD males (*vs*. control males) and while a tendency for an increase was observed in males with the *L*/*L* genotype, this was not significant. The female (control or *S*/*S*) 5-HT2C receptor expression relative to PSD-95 was significantly higher than levels in corresponding male samples (Figure 11).

### (d) 5-HTT distribution in human brain and in immortalized cell cultures

**Figure 12:**
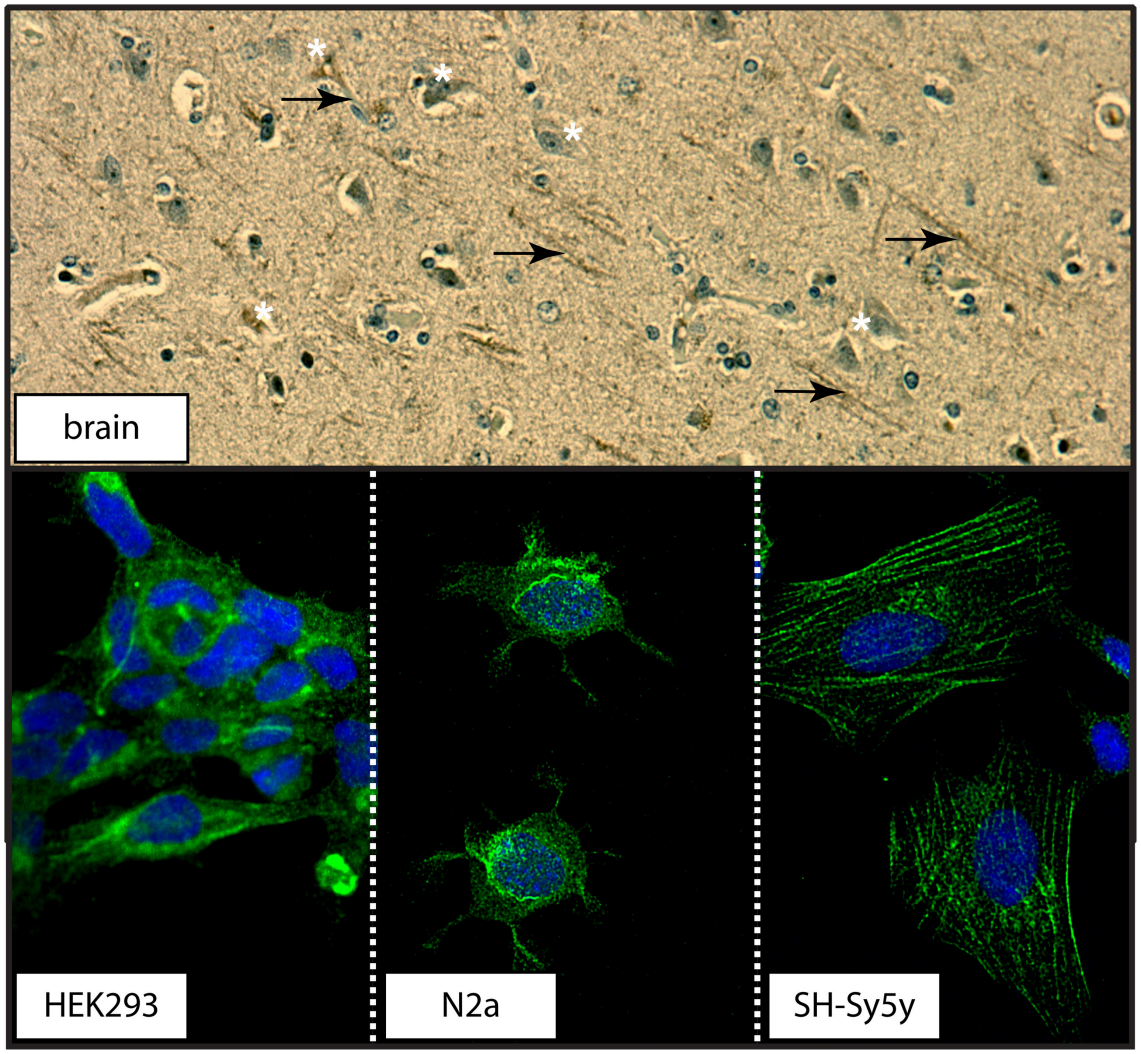
The distribution of 5-HTT in human cortex or in immortalized neuronal-like cell lines. (**top**) The distribution of 5-HTT was demonstrated by DAB-immunohistochemistry in human parietal cortex section. 5-HTT immunodetection appears in both cell bodies (white asterisks) and processes (black arrows). (**bottom**) The distribution of 5-HTT (green) in the HEK293 (human kidney), N2a (mouse neuroblastoma), and SH-Sy5y (human neuroblastoma) cell lines was examined using confocal microscopy (60X magnification). DAPI (blue) was used to counterstain the cell nucleus.

The transport function of the 5-HTT relies on its localization at the plasma membrane (Blakely et al., 1994). Examination of a section of human control brain revealed 5-HTT immunodetection in neuronal cell bodies and processes in human brain (Figure 12). Distinct distribution patterns were also observed in the three cell cultures used: 5-HTT expression was diffuse in human neuronal-like embryonic kidney (HEK 293) cells, perinuclear in mouse neuroblastoma N2a cells, and associated with the cytoarchitecture in human neuroblastoma SH-Sy5y cells (Figure 12).

### (e) 5-HTT, VMAT2, and 5-HT2C receptor expression as a function of APOE ε4 status

**Figure 13:**
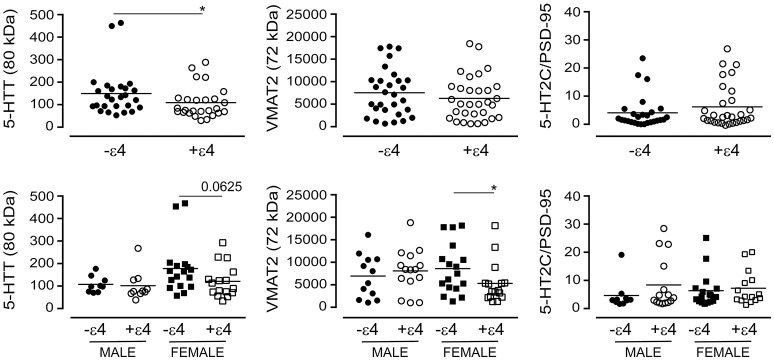
The levels of glycosylated forms of the 5-HTT, VMAT2, and 5-HT2C receptor (relative to PSD-95 expression) were stratified according to the donor's *APOE* ε4 status (i.e., as non-carriers (-ε4) or carriers (+ε4) of the allele). (**bottom**) The data were separated according to the donor's sex *P < 0.05 between indicated groups.

The *APOE* ε4 allele is a significant risk factor for late-onset AD (Poirier et al., 1993). We chose to examine whether the glycosylation profiles we had observed with the donor's diagnosis or 5-HTTLPR genotype aligned with the donor's *APOE* ε4 status. For this analysis, we ignored diagnosis and 5-HTTLPR genotype, and focused strictly on *APOE* ε4 status as a dichotomous nominal variable, i.e., a carrier (+ε4: has at least one ε4 allele) *vs*. non-carrier (-ε4). Levels of the 80 kDa glycosylated form of 5-HTT were diminished in carriers of the ε4 allele in pooled data [i.e., male + female: *P* = 0.0404] and this was reflected by a tendency for a loss of glycosylated 5-HTT in female carriers of the ε4 allele [*P* = 0.0625], but not male carriers [*P* = 0.3947] (Figure 13). There was no influence of ε4 status on the glycosylated form of VMAT2 using pooled data [*P* = 0.3678], but a significant loss of glycosylated VMAT2 was observed in female carriers of the ε4 allele [*P* = 0.0196], but not male carriers of the allele [*P* = 0.6029]. Finally, there was no significant influence of the ε4 allele on the 5-HT2C receptor to PSD-95 ratio whether the data were pooled [*P* = 0.4199] or stratified by sex [male: *P* = 0.2814; female: *P* = 0.2643] (Figure 13).

## Discussion

Altered biogenic amine neurotransmitter metabolism and receptor density in several models of AD are believed to be epiphenomena of the amyloid burden incurred by a given transgene (Szapacs et al., 2004; Liu et al., 2008; Nyarko et al., 2018a). Yet, while cognitive dysfunction and memory loss are certainly characteristics of the later stages of clinical AD, earlier stages of the disease can be associated with non-cognitive, neuropsychiatric phenotypes including depression, irritability, aggressive outbursts, and delusions (Ritchie and Lovestone, 2002). Cognitive impairment often accompanies clinical depression (Emery, 2011) and, not surprisingly, depression is now an acknowledged risk factor for AD, such that it might represent a prodrome for AD-related dementia in certain patient cohorts (Geerlings et al., 2008; Caraci et al., 2010; Wuwongse et al., 2010). Therefore, the potential for overlapping biological mechanisms between depression and AD certainly exists. Depression is commonly associated with a functional deficiency of biogenic monoamine neurotransmitters such as 5-HT.

The *S* allele of the 5-HTTLPR, characterized by a 44 bp deletion, is one of the factors thought to contribute to changes in 5-HT availability and increased risk of depression (Cervilla et al., 2006), cognitive deficits in depressed individuals (Kalska et al., 2013), and cognitive deficits leading to an AD/dementia (Li et al., 1997; Oliveira et al., 1998). Much of this is centered on the original premise that the *S* allele is less transcriptionally active (Heils et al., 1996) and, thus, should lead to a lower expression of 5-HTT (Lesch et al., 1996). While some studies do demonstrate an association between the *S* allele and a loss of *5-HTT* mRNA (Little et al., 1998) and 5-HTT binding capacity (Heinz et al., 2000), others do not support this model (Greenberg et al., 1999; van Dyck et al., 2004; Lim et al., 2006). A recent review suggests that the many factors that can affect 5-HTT expression and PET ligand binding, in combination with the low association between *SLC6A4/5-HTT* mRNA transcript levels and protein expression, is in keeping with the notion that the 5-HTT is dynamically expressed and is highly dependent on post-translational modification(s) (Komorowski et al., 2017). Our current data do not support an influence of the *S* allele on transcription or expression of the 5-HTT gene product, at least in the context of the cortical AD autopsy samples, which corroborates a previous study (Kunugi et al., 2000). In fact, our data appear to indicate a slightly higher level of several 5-HTT species in female carriers of the *S*/*S* genotype, which is in keeping with a report based on (^123^I)-CIT SPECT (van Dyck et al., 2004), but contrasts a report based on [^11^C]-DASB PET imaging in healthy adult males (Bose et al., 2011). Quite unexpectedly, our data do identify differences in the glycosylation status of serotoninergic synaptic markers across the three 5-HTTLPR genotypes, i.e., *S*/*S, S*/*L*, and *L*/*L*.

Glycosylation is a prevalent post-translational modification (Ohtsubo and Marth, 2006) that regulates multiple biological processes such as cell adhesion, signal transduction, and endocytosis. The glycosylation of the 5-HTT is thought to facilitate its proper folding and insertion at the plasma membrane, and to help in protecting 5-HTT against degradation (Blakely et al., 1994) as well as being important for [^125^I]RTI binding (Tate and Blakely, 1994) and 5-HTT dimerization (Ozaslan et al., 2003). Even though it has been suggested that N-glycosylation is not a requirement for 5-HT binding (Blakely et al., 1994; Tate and Blakely, 1994), a lysine-to-asparagine substitution (i.e., K201N) enhances N-glycosylation and expression/stability of the 5-HTT and 5-HT uptake (Rasmussen et al., 2009), whereas deglycosylation has a significant impact on 5-HTT uptake function in human platelets (Launay et al., 1992). Perhaps part of this ambiguity lies with the fact that the glycosylation pattern of the 5-HTT differs depending on the cell type (Qian et al., 1995) or that 5-HTT protein can be expressed at various sites throughout the cell (Huang and Pickel, 2002), as we have also demonstrated herein.

Any change in the capacity for binding/transporting ligand could impact 5-HT signaling. An inverse relation on PET imaging between 5-HTT binding capacity and the presynaptic 5-HT2A receptor density was thought to potentially reflect inter-individual differences in baseline levels of 5-HT (Erritzoe et al., 2010). Yet PET imaging is not currently sufficiently developed to discriminate between 5-HTT species. In our samples, the increased abundance of the post-synaptic 5-HT2C receptor (relative to PSD-95), particularly that of the glycosylated/membrane-associated form in male LOAD samples (*vs*. male control samples) or in males with an 5-HTTLPR *L*/*L* genotype (*vs*. males with a *S*/*S* genotype) does not match any change in 5-HT availability/turnover. In contrast, the tendencies for differences between male and female controls (or male and female carriers of the *S*/*S* alleles) might be a compensatory mechanism (Heiming and Schaser, 2010) following reduced 5-HT availability and/or differences in 5-HT signaling, as indicated by differences in turnover between male and female controls or male and female carriers of the various 5-HTTLPR alleles.

Our observation of sex-dependent biological changes associated with the 5-HTTLPR is not entirely unexpected, given that healthy women have less 5-HTT binding than age-matched men (Jovanovic et al., 2008). Female carriers of the *S* allele also tend to have smaller hippocampal volume (this is relevant to both depression and AD) (Everaerd et al., 2012). Epigenetics (Vijayendran et al., 2012) and splice variants (Murphy and Moya, 2011) might contribute to these phenotypes, yet our work suggests post-translational changes in proteins centered on the 5-HT synaptic junction that align well with 5-HTTLPR genotype.

VMAT2 mediates the uptake of monoamines, including 5-HT, from the cytoplasm into the synaptic vesicle (Henry et al., 1998) and inhibition of VMAT2 by reserpine–and the ensuing vesicular monoamine depletion and loss of regulation of quantal neurotransmitter release–has long been known to induce a depression-like phenotype (Frize, 1954; Iritani et al., 2006). Our observed loss of a glycosylated form of VMAT2 with a diagnosis of AD corroborates the loss of VMAT2 observed in AD (Lehericy et al., 1994) as well as the loss of the 72 kDa glycosylated form of VMAT2 from the vesicle-enriched synaptic fraction of the aging rat brain (Cruz-Muros et al., 2008). Part of this might be a response to 5-HT availability within the synapse, which has been shown to regulate VMAT2 expression in a receptor-independent, but G protein-dependent, manner that allows for the vesicle and vesicular content to adapt to changes in the cellular microenvironment (Holtje et al., 2003; Iritani et al., 2006). Changes in 5-HT availability and/or 5-HT turnover (and post-synaptic 5-HT2C receptor density) that align with either a diagnosis of AD or that are influenced by one's 5-HTTLPR genotype could certainly constitute such a change in the environment.

While a loss in protein glycosylation in the AD brain (Schedin-Weiss et al., 2014) supports patterns of cell death, an increase might be an adaptive survival response (Frenkel-Pinter et al., 2017). Our data suggest a pre-synaptic loss of glycosylation (on 5-HTT and VMAT2), but an increase in post-synaptic (i.e., 5-HT2C receptor) glycosylation. There are reports of pre- and post-synaptic differences in glycosyl transferases e.g., (Hoyte et al., 2002) and there is evidence that 5-HT can trigger a “switch” between glycans/transferases that would favor platelet aggregation (Mercado et al., 2013). Thus, a 5-HT-mediated switch, influenced by a change in 5-HTT function in AD–or with a given 5-HTTLPR genotype–could certainly help to explain concurrent decreased and increased glycosylation of pre- and post-synaptic proteins, respectively. Interestingly, an older study clearly demonstrated that axonal transport and surface expression of glycosylated proteins could be pharmacologically inhibited, yet transport of said proteins and expression in proximal portions of the dendrites was retained, suggesting a difference in vulnerability of the somatic and dendritic protein synthetic machinery (Torre and Steward, 1996). Thus, it is quite possible that an axonal/pre-synaptic loss of glycosylation could occur concurrently with a dendritic/post-synaptic increase in glycosylation.

The negative correlation between VMAT2 and 5-HTT glycosylation in males with a diagnosis of LOAD or in males with a 5-HTTLPR *L*/*L* genotype, but not in corresponding female samples (where any correlation is actually positive), is particularly interesting. Indeed, if the *L*/*L* genotype can lead to differential glycosylation of two important components of the 5-HT synaptic junction in a sex-dependent manner, then this might explain some of the ambiguity surrounding the reported association between the 5-HTT and risk of depression, or response to SSRI treatment, or cognitive status (i.e., AD/dementia). Some of this difference might potentially rely on different types of glycosylation reactions. For example, the loss of N-/O-glycosylation (often capped with sialic acid) might reflect endoplasmic reticulum stress, whereas the increase in O-GlcNAcylation, which involves an initial binding of N-acetylgalactosamine followed by any one of several sugar moieties, might actually be an adaptive cellular response designed to mitigate risk of cell death during endoplasmic reticulum stress (Frenkel-Pinter et al., 2017). This form of glycosylation has been found to protect the AD brain from an accumulation of the neurotoxic β-amyloid peptide (Akasaka-Manya et al., 2010). It is not surprising, therefore, that O-GlcNAc glycosylation is not evident in the hippocampus (a region that is particularly vulnerable during AD) (Hoyte et al., 2002) and that our preliminary data based on a screen of hippocampal samples (*data not shown*) does not reveal any significant changes in glycosylation in response to a diagnosis of AD or to 5-HTTLPR genotype. It is also potentially relevant to our findings that O-GlcNAc glycosylation is post-synaptic (at least in the cholinergic junction of the myofiber) (Hoyte et al., 2002) and that sex-dependent glycosylation has been observed (Knezevic et al., 2010; Ruhaak et al., 2011), with women having significantly more O-GlcNAc glycosylation throughout the lifespan, whereas it tends to increase slowly as men age (Ding et al., 2011).

The *APOE* ε4 allele remains the greatest genetic risk factor for the late-onset/sporadic form of AD (Poirier et al., 1993). Our current analyses reveal a sex-by-*APOE* ε4 status (i.e., as carrier *vs*. non-carrier of the ε4 allele) interaction, with a specific decrease in the glycosylation of the 5-HTT and of VMAT2 in female carriers of the ε4 allele. This proved to be independent of a diagnosis of AD or 5-HTTLPR genotype (as our analyses excluded “diagnosis” or genotype as nominal variables). There were no changes in levels of 5-HT2C receptor (relative to PSD-95), suggesting distinct influences of the ε4 allele and of the 5-HTTLPR genotypes on glycosylation in the 5-HT synaptic junction in our sample set. This could explain, in part, the lack of segregation of the individual 5-HTTLPR alleles or *APOE* alleles in a Brazilian AD cohort, while the combination of these alleles was significantly more frequent in AD patients than in age/sex-matched controls (Nishimura et al., 2005). The MAO-A enzyme, which degrades 5-HT and other biogenic amines, could be contributing to the risk of AD in the Brazilian cohort (Nishimura et al., 2005), although a more recent study could not associate either the 5-HTTLPR genotype or a low-activity MAO-A variant with AD, but did observe an association between the 5-HTTLPR/MAO-A variants and a history of depression (Scholz et al., 2014). A previous study had associated the 5-HTTLPR *S* allele and a high-activity MAO-A variant with a more efficient executive control of working memory performance (Enge et al., 2011). It is interesting that others have found that psychotic manifestations in an AD cohort was associated with the 5-HTTLPR *L* allele, but was not influenced by the *APOE* ε4 allele (Quaranta et al., 2009), yet our sample set did not reveal any changes in dopamine metabolism (often associated with psychosis) with the 5-HTTLPR alleles. In any case, this does support the potential for distinct influences of these two gene risk factors on AD and/or related neuropsychiatric sequelae.

Both increases and decreases (across a range of glycosyl transferases) have been observed in a mouse model of depression and in patients with major depressive disorder (Yamagata et al., 2018), while AD-related neuropathology can also be influenced by glycosylation. Indeed, the amyloid precursor protein (that is cleaved to yield the toxic β-amyloid peptide), tau (that leads to the neurofibrillary tangles), and BACE1/β-secretase and nicastrin/γ-secretase, are all regulated by glycosylation, which can affect signaling mechanism and/or secretase-mediated APP processing and/or clearance of β-amyloid peptides (Schedin-Weiss et al., 2014). We now extend this list to include the 5-HTT, VMAT2, and the 5-HT2C receptor.

Our post-mortem findings likely reflect the progressive course of the disease, making it difficult to extrapolate to earlier stages of the disease progression. Our data strongly suggest that serotoninergic changes and function could contribute to the reported gender-risk of neuropsychiatric symptoms as well as cognitive decline associated with the 5-HTTLPR length polymorphism, yet any risk does not appear to be associated strongly with any overt change in 5-HTT expression. The role of the 5-HTT is clearly not as straightforward as anticipated and any risk of the 5-HTTLPR polymorphism(s) might rely more so on changes in 5-HT signaling associated with transporter (e.g., 5-HTT or VMAT2) glycosylation states, with a sex-dependent pattern potentially complicating the issue. Parenthetically, preliminary results suggest region-dependent profiles, i.e., glycosylation patterns are unaltered in corresponding hippocampal samples. While it is not clear how different glycosylation states might affect PET radiotracer binding and introduce a high degree of variability in any imaging study, or how glycosylation might alter the response to serotoninergic antidepressant drug treatments, it is important to understand that differences in the glycosylation status of the 5-HTT, VMAT2, and 5-HT2C receptor do exist and any one, or combination thereof, could enhance or interfere with 5-HT signaling. These data could also inform on the risk associated with serotoninergic drug usage in depressed patients or early-stage AD, regardless of their cognitive status. Indeed, we have recently shown that individuals with an AD/dementia were almost 3.25 times more likely to have been exposed to an antidepressant drug prior to the onset of AD (Moraros et al., 2017), while a previous study had linked SSRI usage with increased risk of AD/dementia (Kessing et al., 2009).

In summary, the 5-HTTLPR genotype (i.e., *S* and *L* alleles) does not match 5-HTT mRNA and/or protein expression levels. Unexpectedly, the 5-HTTLPR genotype/length polymorphism aligns with distinct sex-dependent glycosylation states of the 5-HTT and other markers of the 5-HT synapse (e.g., VMAT2 and the 5-HT2C receptor). From a purely pathological perspective, our data strongly suggest that AD might follow distinct molecular courses in the male and female brain, with differences paralleling changes in depression-related monoamine systems and risk being influenced differently by 5-HTTLPR length polymorphisms and *APOE* ε4 allele status.

Depression and AD are two important global mental health burdens (Collins et al., 2011). The risk of developing AD/dementia is increased in individuals with a history of depression. In any given year numerous individuals are at risk of developing depression; two-thirds are women. Therefore, it is critical to determine how either of these diseases pivots on the sex of the patient and to acknowledge the possibility of sex-dependent differences in their basic pathobiologies. Such an understanding will inform on optimal pharmacotherapeutic interventions in the clinical context, particularly at early stages, when intervention can still provide long-term benefit.

## Author contributions

JN, MQ, AF, GB, and DM experimental design. JN, MQ, RH, PP, LP, KK, OA, AE, GR, and DM data collection and analysis. All authors prepared the manuscript and editing.

### Conflict of interest statement

The authors declare that the research was conducted in the absence of any commercial or financial relationships that could be construed as a potential conflict of interest.
